# Supplementary figures and images for: Debunking misleading graphs effectively: How vocationally educated young adults perceive graphs
Source: PLoS One. 2026 Feb 9;21(2):e0340100. doi: 10.1371/journal.pone.0340100 (PMC12885246; doi:10.1371/journal.pone.0340100)

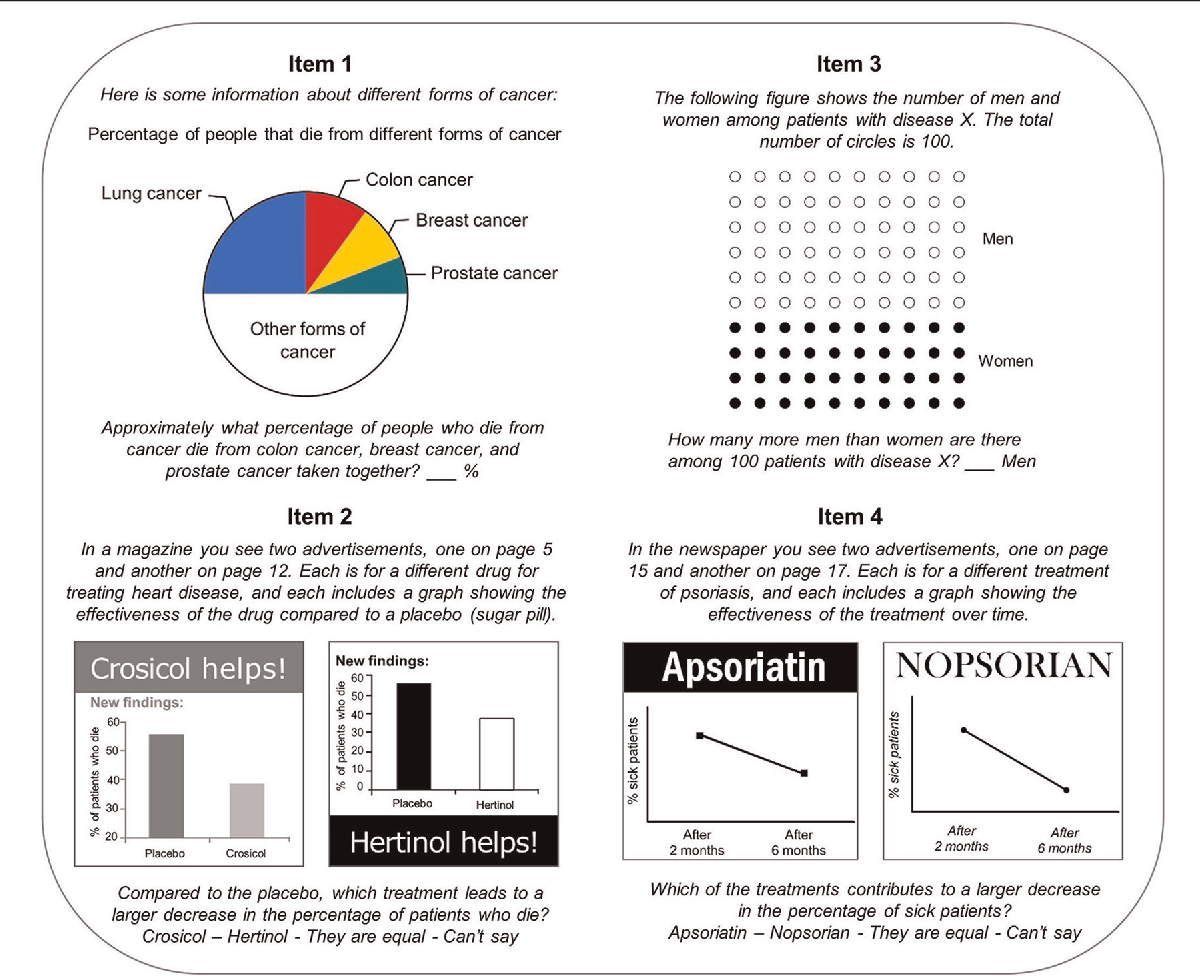

Supplement: S1 Fig — As developed by Okan, Janssen, Galesic and Waters (2019). (TIFF) [file pone.0340100.s001.tiff]
