# Supplementary material for: Debunking misleading graphs effectively: How vocationally educated young adults perceive graphs
Source: PLoS One. 2026 Feb 9;21(2):e0340100. doi: 10.1371/journal.pone.0340100 (PMC12885246; doi:10.1371/journal.pone.0340100)
Supplement: S1 File — Including S1 File Fig. (PDF) [file pone.0340100.s002.pdf]

S1 File. Comparison of data collected at the MBO schools and online via KiesKompas.

While collecting data in the classrooms, we got the impression not all students were filling in the questionnaire seriously. This observation was confirmed by the data where we observed high peaks for 0, 50 and 100 (Fig SB1, left) indicating that they probably did not set the location for the slider with a lot of precision, but put it in the middle of the VAS, where the slider started at default, or moved it to one of the two extremes. These peaks, especially at 50%, are lower or absent in the data collected online via KiesKompas (Fig SB1, right), indicating that this second sample of participants did take more care while filling in the survey and that these data are more trustworthy.

Additionally, in the interviews, we noticed that some graphs or questions were confusing to the participants and the researchers had to give extra explanations, and that students often answered the questions based on how well their expectation on the topic matched with the graph, instead of based solely on the graph itself. Confusion about the question usually happened when the title of a graph was referring to one of the groups shown in the graph, but the question was about the other group and students did not notice this. The participants' opinions were especially influential for the pie chart indicating that 75% of the youth waits with sex until their 18th birthday. This data was often questioned by participants as they expected most adolescents to have sex at an earlier age. Similarly, many did not believe that 78% of clothing allowances are spent only on shoes. For both these contexts, we see a wide spread of evaluations on the VAS (Fig SB1, left), suggesting that either the participants' opinion widely differed, or that some based their answers on their expectation while others solely based it on the information in the graph. After changing the instructions for the online survey and asking participants to ignore their own expectations, the contexts are interpreted more similarly for each graph type (Fig SB1, right).

To improve the questionnaire used in the interviews for use in the online survey, we changed four graph items. We changed the icons of two pictorial area graphs, because in the interviews we noticed they were seen as bars. We changed the question for two pie charts, because they did not match the category that was emphasized in the title of the graph.

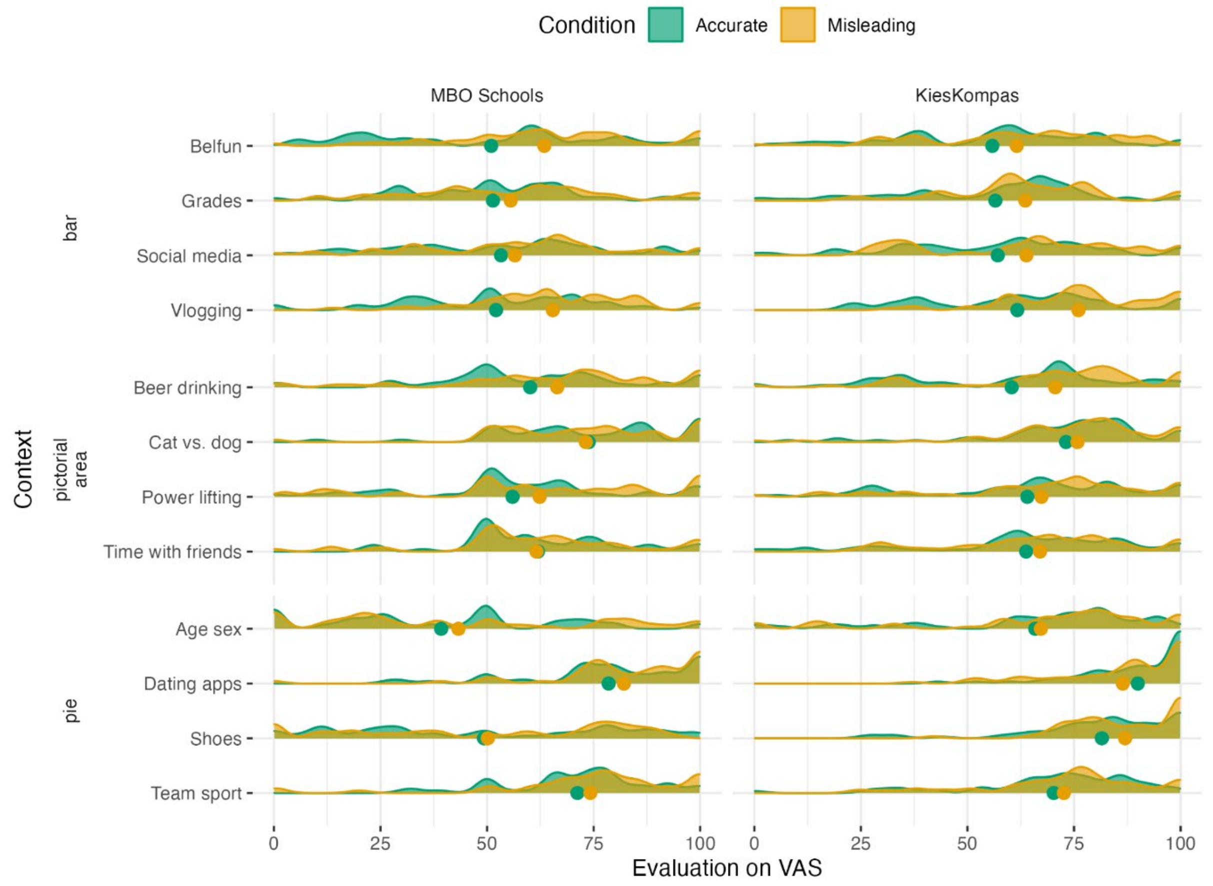

Fig. Density plots showing the distribution of the evaluation scores of the accurate and misleading graphs on the VAS scale at baseline, separate for each context, within the data collected at the MBO schools (left) and online via KiesKompas (right). The dots indicate the mean values per group.
