# Supplementary material for: Debunking misleading graphs effectively: How vocationally educated young adults perceive graphs
Source: PLoS One. 2026 Feb 9;21(2):e0340100. doi: 10.1371/journal.pone.0340100 (PMC12885246; doi:10.1371/journal.pone.0340100)
Supplement: S2 File — Including S2 File Tables 1–3. (PDF) [file pone.0340100.s003.pdf]

S2 File. Analyses manipulation check - Were the graphs indeed misleading? (H1)

In this appendix, the results regarding the research question whether the misleading graphs were indeed misleading are shown. First, *t*-tests were performed for each context separately, with a multiple-testing correction, to check whether there was a significant difference between the mean evaluations of the misleading and accurate graphs. The results are shown in Table 1. To increase power, we also ran the manipulation check with one *t*-test per graph type (bar/pictorial area/pie charts) instead of per context, again with correcting for multiple testing. The results are shown in Table 2.

Table 1. Results of *t*-tests checking whether, at baseline, the mean evaluations (on VAS scale) of the misleading graphs are significantly higher than those of the accurate graphs on the same context.

**Graph type**	**Context**	**Evaluation accurate graph (*M*)**	**Evaluation misleading graph (*M*)**	**Difference in means**	***t***	***df***	***p***
Bar	Belfun	55.83	61.56	5.73	-1.41	123.61	.080
	Grades	56.55	63.58	7.03	-1.99	127.88	.024
	Social media	57.10	63.83	6.73	-1.65	123.19	.051
	Vlogging	61.69	76.05	14.36	-4.21	127.49	**< .001**
Pictorial area	Beer drinking	60.37	70.59	10.23	-2.44	126.88	.008
	Cat vs. dog	73.12	75.80	2.68	-0.80	119.84	.214
	Power lifting	64.04	67.36	3.31	-0.75	124.72	.226
	Time with friends	63.76	67.01	3.25	-0.81	114.70	.211
Pie	Age sex	65.89	67.22	1.33	-0.28	120.08	.391
	Dating apps	89.97	86.44	-3.53	1.41	127.98	.919
	Shoes	81.52	86.98	5.46	-2.04	125.02	.022
	Team sport	70.24	72.61	2.37	-0.69	121.20	.247

Note. Holm-Bonferroni correction was applied to determine whether differences in means were significant. Significant results are indicated in bold.

Table 2. Results of t-tests checking whether, at baseline, the mean evaluations (on VAS scale) of the misleading graphs are significantly higher than those of the accurate graphs of the same graph type.

**Graph type**	**Evaluation accurate graph (*M*)**	**Evaluation misleading graph (*M*)**	**Difference in means**	***t***	***df***	***p***
**Bar**	57.92	65.93	8.01	-4.16	516.32	**< .001**
**Pictorial area**	65.03	70.30	5.27	-2.61	512.46	**.005**
**Pie**	76.61	78.42	1.82	-0.96	517.09	0.169

Note. Holm-Bonferroni correction was applied to determine whether differences in means were significant. Significant results are indicated in bold.

For exploratory purposes, we ran a mixed effects model that includes the misleading condition and graph type, their interaction and the Graph Literacy sum score as fixed effects, and graph contexts and participants as random effects, see results in Table 3.

Table 3. Results of the mixed effects models modelling the evaluations (on the VAS) of the graphs shown at baseline. The model includes fixed effects for whether a graph is misleading or accurate (Misleading?) and for graph type (Graph type), their interaction and the Graph literacy, and with graph contexts and participants as random effects.

**Parameter**	**Categories**	**ß**	***SE***	***t(1502)***	***p***
**(Intercept)**		58.12	4.00	14.51	< .001
**Misleadingness**	Accurate	*Ref.*			
	Misleading	7.77	1.83	4.24	< .001
**Graph type**	Bar	*Ref.*			
	Pictorial area	7.30	5.06	1.44	.149
	Pie	18.77	5.06	3.71	< .001
Interactions:					
**Misleadingness * Graph type**	Misleading, Pictorial area	-3.25	2.59	-1.26	.210
	Misleading, Pie	-5.93	2.59	-2.29	.022
**Graph literacy**		-0.02	0.70	-0.03	.974
