# Supplementary material for: Debunking misleading graphs effectively: How vocationally educated young adults perceive graphs
Source: PLoS One. 2026 Feb 9;21(2):e0340100. doi: 10.1371/journal.pone.0340100 (PMC12885246; doi:10.1371/journal.pone.0340100)
Supplement: S3 File — Including S3 File Tables 1 and 2. (PDF) [file pone.0340100.s004.pdf]

S3 File. Analyses of direct effect of showing a correction (H2-H3).

To determine whether the corrections influence the evaluation of the graphs, the evaluations of the misleading graphs at baseline are compared to those of the same graphs at correction (treatment).
First, a mixed effects model is fitted to study the direct effect of showing a correction, independent of design type (results Table SE1). This model predicts the evaluation score on the VAS scale and includes the misleadingness (misleading vs. corrected), graph type (bar/pictorial area/pie charts) and their interaction as fixed effect, and graph contexts and participants as random effects to accommodate for any random differences between the contexts and participants (Model A). For exploratory purposes, this model is also extended to include the graph literacy scores (Model B).

Table 1. Results of the mixed effects models modelling the evaluations (on the VAS) of the graphs shown at baseline and at correction. The base model includes fixed effects for the misleadingness of the graphs, graph type and their interaction, and graph contexts and participants as random effects (Model A), and is extended with the additional fixed effect of Graph Literacy (Model B).

			**Model A**					**Model B**			
**Parameter**	Categories		ß	*SE*	*t(1551)*	*p*		ß	*SE*	*t(1504)*	*p*
**(Intercept)**			66.26	3.32	19.98	< .001		68.66	3.87	17.74	< .001
**Misleadingness**	Misleading		*Ref.*					*Ref.*			
	Corrected		-10.00	1.77	-5.66	< .001		-9.60	1.79	-5.35	< .001
**Graph type**	Bar		*Ref.*					*Ref.*			
	Pictorial area		3.94	4.58	0.86	.390		3.97	4.58	0.87	.386
	Pie		12.12	4.58	2.65	.008		12.87	4.58	2.81	.005
**Graph literacy**								-1.11	0.82	-1.36	.174
Interactions:											
**Misleadingness * Graph type**	Corrected, Pictorial area		1.11	2.50	0.44	.657		0.79	2.54	0.31	.756
	Corrected, Pie		3.87	2.50	1.55	.122		3.16	2.54	1.25	.213

Additionally, to answer H3, we fitted one mixed effects model to test the difference between the evaluations of corrections in clean and full-design (results in Table 2). This model is fitted only on the data from the corrected graphs and includes the correction design (clean vs. full), the graph type and their interaction as fixed effects, and again context and participants as random effects.

Table 2. Results of the mixed effects models modelling the effect of the correction design on the evaluations (on the VAS) of the graphs shown at correction. The model includes fixed effects for the correction design (clean vs. full-design), graph type and their interaction, and graph contexts and participants as random effects.

**Parameter**	**Categories**	**ß**	***SE***	***t(1551)***	***p***
**(Intercept)**		57.16	3.30	17.34	< .001
**Correction design**	Clean	*Ref.*			
	Full	-1.76	3.11	-0.57	.571
**Graph type**	Bar	*Ref.*			
	Pictorial area	7.68	4.27	1.80	.072
	Pie	17.43	4.27	4.09	< .001
Interactions:					
**Correction design * Graph type**	Full, Pictorial area	-4.89	3.56	-1.37	.170
	Full, Pie	-2.58	3.56	-0.72	.469
