# Supplementary material for: Debunking misleading graphs effectively: How vocationally educated young adults perceive graphs
Source: PLoS One. 2026 Feb 9;21(2):e0340100. doi: 10.1371/journal.pone.0340100 (PMC12885246; doi:10.1371/journal.pone.0340100)
Supplement: S4 File — Including S4 File Tables 1–3, and S4 File Fig. (PDF) [file pone.0340100.s005.pdf]

## S4 File. Models fitted to determine the learning effect of showing a correction.

To determine whether showing a corrected graph generally has an influence on the evaluation of new misleading graphs, the evaluations of new misleading and accurate graphs, shown after the corrections, are compared. The mixed effects model includes as fixed effects whether a graph is misleading or accurate ("Misleadingness"), graph type and their interaction, and context and individuals are included as random effects in the model. The results are shown in Table 1, New. For comparison with the original misleading effect of a misleading graph, a similar model is fitted for the misleading and accurate graphs at baseline. These results are also shown in Table 1, Baseline.

**Table 1.** Results of the mixed effects model modelling the evaluations (on the VAS) of the graphs shown before (Baseline) and after (New) corrections. The models includes fixed effects for whether a graph is misleading or accurate (Misleadingness), graph type and their interaction, and graph contexts and participants as random effects.

| | | | **Baseline** | | | | | **New** | | | |
|---|---|---|---|---|---|---|---|---|---|---|---|
| **Parameter** | Categories | | ß | *SE* | *t(1551)* | *p* | | ß | *SE* | *t(1504)* | *p* |
| **(Intercept)** | | | 57.71 | 3.61 | 15.97 | < .001 | | 48.01 | 6.99 | 6.87 | < .001 |
| **Misleadingness** | Accurate | | *Ref.* | | | | | *Ref.* | | | |
| | Misleading | | 8.43 | 1.81 | 4.67 | < .001 | | 6.01 | 1.90 | 3.17 | .002 |
| **Graph type** | Bar | | *Ref.* | | | | | *Ref.* | | | |
| | Pictorial area | | 7.52 | 5.05 | 1.49 | .137 | | 17.40 | 9.85 | 1.77 | .078 |
| | Pie | | 19.09 | 5.05 | 3.78 | < .001 | | 19.10 | 9.85 | 1.94 | .053 |
| **Graph literacy** | | | | | | | | | | | |
| ***Interactions:*** | | | | | | | | | | | |
| **Misleadingness * Graph type** | Misleading, Pictorial area | | -3.54 | 2.55 | -1.38 | .167 | | -1.04 | 2.68 | -0.39 | .698 |
| | Misleading, Pie | | -7.00 | 2.55 | -2.74 | .006 | | -8.24 | 2.68 | -3.07 | .002 |

To study the difference in the learning effect of the two correction designs, in Fig SF1 we plotted the distributions of the evaluations of the new misleading and accurate graphs that were shown after participants saw the corrections in one of the two designs. In Table 2 these differences are tested with a mixed effects model including all these factors as fixed effects in the model, i.e. whether the graphs were misleading, the correction design, and the graph type, and their interactions, and the context and individual as random effects.

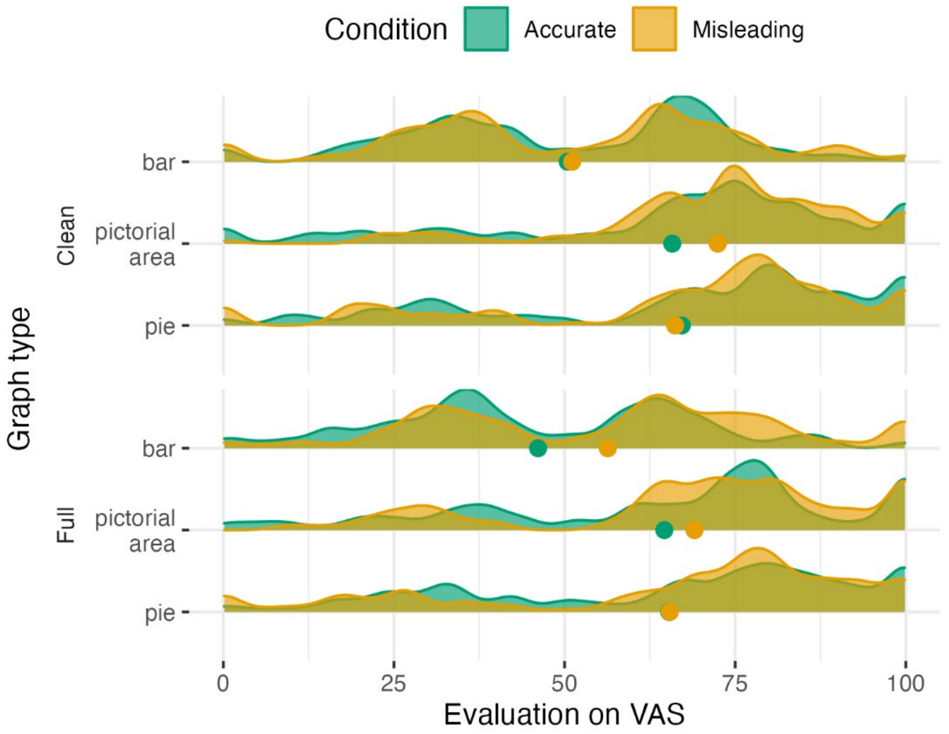


**Fig 1.** Density plots showing the distribution of the evaluation scores of the new accurate and misleading graphs after corrections were shown, separate for each graph type and split depending on the correction design that participants were shown. The dots indicate the mean values per group.

**Table 2.** Results of the mixed effects model modelling the evaluations (on the VAS) of the new graphs shown after corrections of either a clean or full design. The model includes fixed effects for whether a graph is misleading or accurate (Misleadingness), correction design, graph type and their interactions, and graph contexts and participants as random effects.

| **Parameter** | **Categories** | **ß** | ***SE*** | ***t(1545)*** | ***p*** |
|---|---|---|---|---|---|
| **(Intercept)** | | 50.33 | 7.15 | 7.04 | < .001 |
| **Misleadingness** | Accurate | *Ref.* | | | |
| | Misleading | 0.91 | 2.76 | 0.33 | .743 |
| **Correction design** | Clean | *Ref.* | | | |
| | Full | -4.38 | 2.87 | -1.53 | .127 |
| **Graph type** | Bar | *Ref.* | | | |
| | Pictorial area | 15.67 | 10.06 | 1.56 | .119 |
| | Pie | 17.65 | 10.06 | 1.76 | .079 |
| *Interactions:* | | | | | |
| **Misleadingness * Correction design** | Misleading, Full design | 9.63 | 3.78 | 2.55 | .011 |
| **Misleadingness * Graph type** | Misleading, Pictorial area | 5.32 | 3.90 | 1.36 | .173 |
| | Misleading, Pie | -3.48 | 3.90 | -0.89 | .372 |
| **Correction design * Graph type** | Full design, Pictorial area | 3.26 | 3.78 | 0.86 | .388 |
| | Full design, Pie | 2.73 | 3.78 | 0.72 | .471 |
| **Misleadingness * Correction design * Graph type** | Misleading, Full design, Pictorial area | -11.98 | 5.35 | -2.24 | .025 |
| | Misleading, Full design, Pie | -8.97 | 5.35 | -1.68 | .094 |

Finally, as we observed that the evaluations of both the misleading and accurate graphs decreased after participants had seen the corrections, we also fitted a model to compare the evaluations before and after corrections. The mixed effects model includes the stage (before vs. after seeing corrections), whether the graphs are misleading and the graph type as fixed effects, and the contexts and individuals as random effects. Results are shown in Table 3.

**Table 3.** Results of the mixed effects model modelling the evaluations (on the VAS) of the graphs shown before and after corrections. The model includes fixed effects for at what stage in the study the graph was shown (at baseline vs. after corrections), whether a graph is misleading or accurate (Misleadingness) and for graph type, and graph contexts and participants as random effects.

| **Parameter** | **Categories** | **ß** | ***SE*** | ***t(3112)*** | ***p*** |
|---|---|---|---|---|---|
| **(Intercept)** | | 58.21 | 4.42 | 13.17 | < .001 |
| **Stage of study** | Baseline | *Ref.* | | | |
| | After correction | -7.39 | 4.37 | -1.69 | .091 |
| **Misleadingness** | Accurate | *Ref.* | | | |
| | Misleading | 3.92 | 0.76 | 5.17 | < .001 |
| **Graph type** | Bar | *Ref.* | | | |
| | Pictorial area | 11.32 | 5.35 | 2.11 | .035 |
| | Pie | 15.29 | 5.35 | 2.86 | .004 |
