# Supplementary material for: Debunking misleading graphs effectively: How vocationally educated young adults perceive graphs
Source: PLoS One. 2026 Feb 9;21(2):e0340100. doi: 10.1371/journal.pone.0340100 (PMC12885246; doi:10.1371/journal.pone.0340100)
Supplement: S1 Table — (PDF) [file pone.0340100.s006.pdf]

**SC1 Table. Codebook for the thinking-aloud task.**

| Category | Code | Description | Example |
|---|---|---|---|
| **Graph type*** | Pictorial area graph | - | - |
| | Bar graph | - | - |
| | Pie chart | - | - |
| **Correctness*** | Accurate | - | - |
| | Misleading | - | - |
| **Measure time*** | Baseline | - | - |
| | Correction | - | - |
| | New | - | - |
| **Focus** | Reference to area/bar/pie | Participant refers to the bar(s), piece(s) of the pie, or the area(s). | Participant# 6: You can also see in the graph [points to bars] that it is more than double, so this fits in there twice. |
| | Reference to embellishment | Participant refers to the elements in the graph that are not essential to communicate the data but are additional design elements (also 'color'). | PP# 10: […] this font, it is a bit distracting. They make it a lot more fun than it has to be. |
| | Reference to topic | Participant refers to what the graph and its data are about (also 'context'). | PP# 7: Well, we live in a society that is very transparent, so I wouldn't think people would wait, no. |
| | Reference to values/numbers | Participant refers to exact data points, percentages, or amounts that are displayed in the graph or can be deduced from them. | PP# 8: I see that it should be about 40.000 more, approximately. |
| **Evaluation** | Clearness | Participant refers to the clearness of the graph (also 'complicated') or how it interferes with their attention (also 'distracting'). | PP# 10: What really helps now is that the background is not so distracting, it is very soft. |
| | Credibility | Participant refers to the degree to which they find the presented data credible (also 'untrue'). | PP# 7: Eighteen-year-olds drink less and less glasses of beer per week. I doubt that. |
| | Difference | Participant refers to the difference between datapoints (also 'calculation'). | PP# 2: Well, I think the difference is quite okay, also because people can have |

| | | | |
|---|---|---|---|
| | | | more cats than dogs. |
| | Severity | Participant refers to how they feel about the topic. | PP# 2: Well, I think it is a lot. Because it [violation of privacy laws] should actually be zero! |
| **Deceit awareness*** | Level 1: Misleading element spotted | Participant refers to the size-ratio of the two areas in the chart, the cut-off y-axis in the bar graph, or the 3D effect used in the pie chart. | PP# 10: Yes, it would have been better to show it [the pie chart] from the top, and not like half flat [in 3D]. |
| | Level 2: Deceit recognized | Participant declares that the spotted misleading element (level 1) (intentionally) affects their evaluation of the difference between the data points. | PP# 2: Yes, so what I just said, that graph starts at fourteen, so it seems like this is almost a third of the graph, making it seem like a big difference, but actually I think… it is only, it is six euros, so it is a big difference but not as big as they want you to believe. |
| **Strategy** | | Participant refers to how they read the graphs. | PP# 2: Yes, I make a scan first. |

* Exclusive category codes
