# Supplementary material for: Debunking misleading graphs effectively: How vocationally educated young adults perceive graphs
Source: PLoS One. 2026 Feb 9;21(2):e0340100. doi: 10.1371/journal.pone.0340100 (PMC12885246; doi:10.1371/journal.pone.0340100)
Supplement: Attachment — Submitted filename: Comments to Authors.pdf [file pone.0340100.s007.pdf]

Review

Debunking misleading graphs effectively: how vocationally educated young adults perceive graphs

Paper summary

This paper presents an experimental study of graph reading and interpretation in the niche area of vocationally educated students. The methodology involves a mixed methods design with interviews and a survey. Despite a well justified and numerous sample, the authors did not present their findings and discuss their insights well.

Positive Aspects

The research addresses an interesting and relevant topic with practical implications and does so through a compelling combination of qualitative and quantitative methods. The discussion highlights some valuable insights and these aspects would deserve more space in terms of practical implications and applications. In addition, the scope and choice of targets show sensitivity to the social relevance of the issue and the impact of the findings.

Limitations

The reviewed paper suffers from lack of precision and organization in exposition, not proper references and context for strong claims. Unclear presentation of results and missing details hinder readability and comprehension of the study conducted.

Suggested: Major revisions

Overall comment

I found this paper quite difficult to review. In fact, the authors have carried out an interesting study, but they haven't managed to include adequate information and organize their presentation effectively. I am not sure if I have understood well in detail the conditions and variables manipulated, and this affects the final presentation of the results, making them difficult to appreciate.

There are some concerns about the depth of presentation and the use of statistical tests, but I hope this is just '*behind the scenes*' work that is not mentioned and can be addressed in the revision without changing the main outcome of the work. In this connection, for many points the responsibility for interpretation and understanding is left to the reader which could lead to misunderstandings.

I would like to be completely transparent: the paper is poorly written and lacks proper organization, giving the impression that it was rushed and completed without sufficient attention to detail. The presentation of the data lacks of crucial elements, making it difficult to follow or evaluate the work. Unfortunately, in its current form it does not meet the required standards and should be rejected. However, you have an interesting idea and a solid sample, with over 100 responses and 10 interviews, which has the potential to lead to a publication that other scholars will refer to and cite.

If you have the resources for open access and data sharing, don’t let rushed writing be the reason for rejection. In the following lines, I’ve highlighted many points for improvement, but I strongly recommend taking the time to rewrite from scratch at least the key sections of the results and methods (specifically variables and design).

Comments and Improvements Suggestions

Lack of precision and coherence in the presentation. The paper seems to lack coherence due to paragraphs really detailed in contrast to others (maybe even more important) too vague. In the same line, the tone ranges from narrative with many excursus to vague and sharp. Precision is also sometimes lacking in the language.

Lack of adequate references. Many sentences, even strong ones, are not well argued or lack references as well as appropriate inclusion in the relevant literature.
Related work is discussed, but this does not seem to be enough to present a project that fits well into this research framework. Also, the findings should be placed more in the context of this existing literature.

Variables, Statistical Analysis and Quantitative Results.

- I find difficult to keep track of the variables mentioned and manipulated. It would be useful for the contextualisation of your results to have a better presentation (even with a table or a simple scheme) of all the variables, especially when dealing with interactions between them.
- In many points, but especially in the quantitative aspects (methods, results, graphs, etc.), your paper does not clearly present all the necessary elements, leading the reader to interpret or imagine what could have been the work behind it.
- It seems that the authors used a t-test for the analysis, but the lack of details ("*several statistical methods*") makes it impossible to evaluate their choice.
- The quantitative results are presented with a few values and a density plot that lack context and completeness: statistics are not mentioned and the value of an observed difference is without a standard deviation and a p-value for statistical significance.
- The density plot reported in the results section lacks a proper description, even to be addressed qualitatively.
- "*To increase the power, instead of testing for each context, we can combine the graphs and only test for an effect of the misleading graphs within each graph type, which situation is also visualized in Figure 5 (right)*" (p. 21/below figure 5) needs to be better explained addressing pros and cons of your choice. This aspect could be easily fixed by working on

the variables presentation. Unfortunately, at this point of the reading the elements mentioned but not explained can hinder proper results reading.

Qualitative Methods. A more detailed description of the methodology with appropriate references would be helpful to better frame the results: “*open and axial coding*” could be detailed with the type of framework you referred to. There are further questionable aspects: were interviews recorded? If not, why not and what was done to ensure the representativeness of the data? Were any strategies employed to ensure data validity?

Exposition. Although the paper structure is appropriate, the content is not well organized, making it difficult to navigate it smoothly, even within the paragraph. Both the introduction and the literature review could be better structured (e.g. first mention the characteristics you are going to describe and then move on to each one). This type of writing should be as much predictable as possible to make reading easier.

- I would merge paragraphs 2.1, 2.2 and 2.3 in a single one in order to have a stronger context with less lines.
- In this connection, I would change 3.1 “*Study Design*”: this creates a kind of Easter egg when you get to the end of the paragraph. Why not say that the initial plan failed due to non collaborative participation and you exploited this experience as a pilot to refine your study design? On the one hand it helped to refine the methodology, but on the other it should not get so much space.
- The description of why you chose this specific target is quite redundant: I would keep it only in the method for just mentioning it in the limitations (see specific note for limitations).
- Maybe moving 4.1.1 (*Demographics*) in the sample/participants section: first you describe the population you are interested in and then you present the composition of the sample you recruited. Add mean and standard deviation for age and other measures.
- I would suggest better integrating the quotes from interviews to support your argument, even smaller pieces but within the paragraph in the points in which you mention them.

Limitations Limitations go beyond the generalizability motivated by the specific population targeted. Interactions between the variables should be acknowledged, as well as the potential bias

of representation in the topic. I would have expected a more in-depth discussion of the qualitative methodology as well as bias and variables interactions which are pivotal in this study.

Missing Definitions and Descriptions Some of the descriptions are too much on a high level making it difficult to be completely sure of what you are referring to. If there are standards or conventions please cite, instead add at least a very short definition. Especially with strong statements, please consider to be more specific. For example "*Hence, from these observations we can conclude that* ***indeed, generally,*** *the misleading bar and pictorial area graphs were misleading, but* ***some contexts*** *resulted in more differences than others.*" (p. 21/below figure 5) is an important conclusion but it lacks completeness.
