# Supplementary material for: Debunking misleading graphs effectively: How vocationally educated young adults perceive graphs
Source: PLoS One. 2026 Feb 9;21(2):e0340100. doi: 10.1371/journal.pone.0340100 (PMC12885246; doi:10.1371/journal.pone.0340100)
Supplement: Attachment — Submitted filename: Response to Reviewers.docx [file pone.0340100.s009.docx]

**Debunking misleading graphs effectively:
how vocationally educated young adults perceive graphs**

Response to reviews-letter

| **Editor** | | |
| --- | --- | --- |
| E.1 | Please ensure that your manuscript meets PLOS ONE's style requirements, including those for file naming. | *We have changed the file names to meet PLOS ONE’s style requirements.* |
| E.2 | Thank you for stating the following financial disclosure:  “This research was supported by Leiden University Fund (<http://www.luf.nl>, LUF Lustrum Grant 2020, W20719-1-LLS).”  Please state what role the funders took in the study.  If the funders had no role, please state: "The funders had no role in study design, data collection and analysis, decision to publish, or preparation of the manuscript."  Please include this amended Role of Funder statement in your cover letter; we will change the online submission form on your behalf. | *We have included the amended Role of Funder statement in our cover letter as requested:*  *“This research was supported by Leiden University Fund (*[*http://www.luf.nl*](http://www.luf.nl)*, LUF Lustrum Grant 2020, W20719-1-LLS). The funders had no role in study design, data collection and analysis, decision to publish, or preparation of the manuscript.”* |
| E.3 | Please note that your Data Availability Statement is currently missing the direct link to access each database. If your manuscript is accepted for publication, you will be asked to provide these details on a very short timeline. We therefore suggest that you provide this information now, though we will not hold up the peer review process if you are unable. | *The collected quantitative data and transcripts of the interviews are stored and publicly available at Open Science Framework (*[*https://osf.io/gcmf2/?view_only=aee14096a719424c88ac247dfe94a670*](https://osf.io/gcmf2/?view_only=aee14096a719424c88ac247dfe94a670)*). The data of the interviews can be found in the folder “Interviews – Transcripts and Data”, and the survey data can be found in the folder “Surveys – Data and analysis code / KiesKompas / Data”. Unfortunately, OSF does not provide direct links to the data stored in this project.* |
| E.4 | We note that Figures 1 left and 4 top in your submission contain copyrighted images. All PLOS content is published under the Creative Commons Attribution License (CC BY 4.0), which means that the manuscript, images, and Supporting Information files will be freely available online, and any third party is permitted to access, download, copy, distribute, and use these materials in any way, even commercially, with proper attribution. | *We have replaced Figures 1 left and 4 top by copyright free images.* |
| **Reviewer 1** | | |
| R1.0 | […] the authors mention that they struggled recruiting enough participants for the online questionnaire and that participants during the thinking-aloud tasks seemed more motivated and “very open and honest in their answers”. Therefore, the qualitative interviews appear to be well suited for vocationally educated people who are still underrepresented in research. This choice was a big asset of the paper which is very well written and is an interesting and original contribution to the existing literature. The relevance of the investigation of vocationally educated adults (an underrepresented group in research) is also clearly highlighted. | *Thank you for your kind words.* |
| R1.1 | Introduction; paragraph 2. The authors write that the graphs in a previous study were tested “in a clean experimental set-up” and in the current study in a “more realistic setting”. In which ways was the present study a more realistic setting than the previous clean setup? Data was gathered from participants online in an online questionnaire. How was this more realistic? And also the thinking-aloud interviews seem to have taken place in a rather experimental setting. Please elaborate on this with a short example. | *Indeed, it wasn’t as much the set-up as the design and the topics of the graphs that were closer to the type of graphs that the participants would encounter on a daily basis. We have changed “setting” to “approach” (p.3).* |
| R1.2 | Theoretical framework; 2.3; paragraph 3. Please specify here what the “clean design” encompasses. It is explained later on in the text (for example in section 3.3.3), but a short elaboration here with an example would be useful. | *We have added a short description of clean design: “that minimizes use of ink and unnecessary design elements” (p.6).* |
| R1.3 | Methods; 3.1; paragraph 1. How big were the student groups on average and how many teachers were involved for each class? If there were six different teachers and one class for each teacher, certain teacher-specific differences might influence the outcome. Furthermore, if the classes were very large this could also affect the outcomes. | *We have included the amount of teachers and the range of group size in section 3.1 (p.8).* |
| R1.4 | Methods; 3.1; paragraph 2. How were the students for the thinking-aloud interviews chosen? Careful not to consider them to be representative for the entire class because if they volunteered, their motivation for participation may already be higher than for the other students who were also mentioned not to have taken the questionnaire seriously. | *We have added “voluntarily” to the sentence (p.8).* |
| R1.5 | Methods; 3.1; paragraph 4. The authors mention that not all participants were taking the questionnaire seriously. In addition to the reasons given by the authors, this might also be because the students seem to have been somewhat forced to participate in the questionnaire. Even if they could refuse their approval for data collection, they were still forced to complete the questionnaire which is another reason why the decision of the authors to discard these data was justified. | *Thank you for the suggestion. We have added to the sentence “possibly also due to the fact that filling in the questionnaire was a mandatory part of the guest lecture” for more clarification (p.9).* |
| R1.6 | Methods; 3.2; paragraph 1. It was not clear at first that the student data was discarded completely and new participants independent from the classrooms were recruited. I had the impression at first that a new data collection with the same students was set up. Please clarify this here or even in the section above. This might be cleared up by mentioning the classroom data was used as a sort of piloting and testing of the study. | *Thank you for the suggestion. We have added the sentence: “The data collected in the classrooms we found to be not suitable for further analysis (see below for further details) and was treated as a pilot study to further refine the questionnaire and set-up.” (p.8).* |
| R1.7 | Methods; 3.2; paragraph 1. Please report on the gender and age distribution of the participants in the main text. Also specify how the 10 students for the qualitative interviews were recruited with which inclusion or exclusion criteria. | *We have added the gender and age distribution (p.10). Also see response to R1.4.* |
| R1.8 | Methods; 3.3.2; paragraph 1. What are the “predefined protocols” mentioned that were used during the thinking-aloud task? Were these the same as the evaluation questions mentioned later? | *We have added the sentence: “(ask: what are you looking at/what goes on in your mind?)” (p.11). Not linked to the evaluation questions.* |
| R1.9 | Methods; 3.3.3; paragraph 1. Was the fictional information which was used to create the graphs surprising to the participants or was it following common expectations, i.e. what a majority of people believe or expect to be true? Graphs containing surprising information that contradicts their own expectations might spark higher interest in participants, tempting them to look at the graph more closely and this might aid in them spotting misleading features. Graphs, however, that depict a common belief might not be looked at in that much detail and misleading features in these might be more easily overlooked. Please specify which form of fictional information was used and whether this might have had an impact on the results. | *Good argument indeed. We tried to avoid surprise by staying close to reality. We have added the sentence :” The data was fictional but close to reality to make the content of the graphs credible and to avoid provoking surprise that might influence the reading process.” (p.11).* |
| R1.10 | Results; Throughout the results section and the Discussion section please specify how many participants mentioned something or used a certain combination instead of just writing “most students” (p.16), “most often” (p. 18), “most participants” (p.25), “many more participants” (p.27), “many occasion” (p.27). Report the actual numbers or percentages in brackets in the text. | *This study used a mixed methods approach. We report actual numbers and percentages for the quantitative data, but stay away from these for most of the qualitative data to avoid a statistical perspective on these data. To this goal we chose to describe trends rather than reporting amounts.* |
| R1.11 | Results; 4.1.2; paragraph 1. In the first quote it was not clear to me whether the participant was reading the instructions or commenting on the graph. It sounds like reading the instructions so this quote does not seem to contribute any meaning to how the participant was evaluating the graph. | *We have placed the reference to quote 1 to an earlier sentence to clarify that it is an example of how the participants read aloud to demonstrate what they were looking at (p.19).* |
| R1.12 | Results; 4.1.3; paragraph 1. The authors write: “In the interviews all students were shown the clean design correction”. Does this mean they never saw the full design correction and only the clean design corrections? Or did they see both? But then again the participants were commenting on how boring the clean design was but not the full design correction? Please explain. | *Please note the difference between the clean/full design graphs and the clean/full design corrections of the graphs. The students were shown full design graphs, but the corrections were shown in clean design. We have added some words to explicate this (p.19).* |
| R1.13 | Discussion; 5.1; paragraph 1. There is an extra open bracket after the references [14][16][2]. | *Thank you for pointing that out – is now removed.* |
| R1.14 | Discussion; 5.4; paragraph 1. The authors claim that the results found for vocationally educated students are “generalizable to a large group of people” because they take up the biggest share in Dutch society. This fact may make the results more relevant to a large majority, but not more generalizable to the whole. The authors even state below that the participants in their study had much higher graph literacy scores than what they had expected to find. This is an indication that maybe their participants were even an exception within the group of vocationally educated people and might not even represent this group perfectly. So it is not possible to generalize from this specific group to a larger majority. | *Thank you for pointing that out. We have changed the sentence to: “[…] making our results highly relevant when considering possible graph deceit among the Dutch population.” (p.32).* |
| R1.15 | Discussion; 5.4; end of paragraph 3. The authors state that KiesKompas was struggling to reach the required number of participants. Any suggestions why this was the case? This was confusing to me because in the Methods section the authors claimed that the right number of participants was found in the end. How did KiesKompas end up collecting the right amount of data in the end and how did they contact and reach participants in the first place? Please add a short explanation of how this collection service works. | *We have added the sentence: “A possible explanation is that this group is not to keen on or interested in participating in research studies. More research is needed for more clarify on this issue.” (p.33).* |
| **Reviewer 2** | | |
| R2.0 | This is an interesting study that seems unlikely to be done, especially because the main question seems to be so self-evident. If one gets access to a set of data via some graphical illustration, then it seems obvious that the graphs being misleading also leads to a distorted view of the data. Hence, it would not be worth simply investigating such an obvious question. However, the authors go one step further and then ask, how graph correction could be most effectively done to mitigate this distortion effect. As such, there are valuable practical implications of this study that are well worth publishing.  Overall, this is certainly an intriguing project and I thank the authors for their research. | *Thank you for your kind words.* |
| R2.1 | Why exactly was this population selected for the present study? After all, misleading graphs are a problem for all of us. What is the benefit of the think-aloud task over other qualitative methods that might also have been helpful? It seems not surprising that correcting misleading graphs eventually were shown to be less misleading, and also that there might be a learning curve at hand. Given the intuition that this “is not news”, how are these results relevant vis-à-vis both this basic intuition and the existing literature? In other words, what makes these findings so relevant, especially since there was no difference between the specific type of the correction? I believe further strengthening on these arguments might help strengthen the case for this project, since it is not so clear otherwise why it should be relevant given the unsurprising nature of the results. After all, one could argue, that “everybody knows that we can benefit from correction of misleading graphs, not just young adults, and there is no value in a study for this”. This does not say that the study is not needed, but that it is important to make a clear case right from the beginning up until the end of why this study is necessary and relevant. | *In the introduction, paragraph 3, we explain why we chose to select vocationally educated young people.*  *Other qualitative methods could have been useful as well, but as in all studies, our time and resources were limited and the talk aloud task gave us the data we were looking for.*  *Indeed we did not find an effect between the full and clean design corrections, which was only part of our inquiry. We believe the relevance of this study lies more in peek inside the minds of this underrepresented group of Dutch society than in the proof for a greater effect of either design options.*  *We believe these arguments are well discussed throughout the paper, for instance in sections 2.2 and 5.4.* |
| R2.2 | I am not aware how this pertains to the journal’s guidelines, but I would suggest to put the links in the methods section either in the footnotes or as a regular reference. | *Thank you for the suggestions. We will discuss with the journal how to handle the links.* |
| R2.3 | Please check how a study us usually outlined: You described the hypotheses in the “analysis” chapter. This is too late. The goals, research questions and hypotheses need to be elucidated right at the beginning (or, somewhere in the introductory chapter). Introducing them in the methods, analysis or discussion chapter would be too late, since it must tether the whole project to what one actually wants to investigate. | *We have added the sentence “This study addresses the question of how vocationally educated young adults interpret misleading graphs and what design options can best guide the process of seeing through the deceit.” at the end of the introduction (p.3).* |
| R2.4 | Since the paper discusses misleading graphs, it would be helpful to readers to actually see some illustrations of what you mean by misleading graphs and correct graphs. Likewise, it would be helpful to have some visualization of the study design. | *We fully agree. The manuscript includes many example graphs and a visualization of the study design. The figures are uploaded separately from the manuscript, as requested by PLOS ONE. In the Method section we included a new Figure (5 a.-c.) to further visualize the study design.* |
| **Reviewer 3** | | |
| R3.0a | The research addresses an interesting and relevant topic with practical implications and does so through a compelling combination of qualitative and quantitative methods. The discussion highlights some valuable insights and these aspects would deserve more space in terms of practical implications and applications. In addition, the scope and choice of targets show sensitivity to the social relevance of the issue and the impact of the findings. | *Thank you for your kind words.* |
| R3.0b | The reviewed paper suffers from lack of precision and organization in exposition, not proper references and context for strong claims. Unclear presentation of results and missing details hinder readability and comprehension of the study conducted. The paper seems to lack coherence due to paragraphs really detailed in contrast to others (maybe even more important) too vague. In the same line, the tone ranges from narrative with many excursus to vague and sharp. Precision is also sometimes lacking in the language. Many sentences, even strong ones, are not well argued or lack references as well as appropriate inclusion in the relevant literature. Related work is discussed, but this does not seem to be enough to present a project that fits well into this research framework. Also, the findings should be placed more in the context of this existing literature. | *Thank you for your extensive feedback. We have listed your comments and our actions taken for improvement below.* |
| R3.1a | **Variables, Statistical Analysis and Quantitative Results**  I find difficult to keep track of the variables mentioned and manipulated. It would be useful for the contextualisation of your results to have a better presentation (even with a table or a simple scheme) of all the variables, especially when dealing with interactions between them. | *Thank you for your suggestion. We noticed some inconsistencies in our naming of the variables and fixed this.*  *Additionally, we have added visualizations of the variables involved in the hypotheses that are tested, in the new Figures 5a.-c. to help readers understand what is tested.* |
| R3.1b | In many points, but especially in the quantitative aspects (methods, results, graphs, etc.), your paper does not clearly present all the necessary elements, leading the reader to interpret or imagine what could have been the work behind it. | *Thank you for pointing this out. We have clarified the specific points that you mentioned and explained our changes below. We hope that this helps the readers to understand the details of the analysis. Note that many statistical tests were done, but their results are primarily presented in the appendix to avoid an overload of information in the main text. In the main text we focus on visualizing the data via density plots which usually already give the answer to a hypothesis. The statistical tests are given less attention in the main text as their only purpose is to verify the observations in the data visualizations.* |
| R3.1c | It seems that the authors used a t-test for the analysis, but the lack of details ("*several statistical methods*") makes it impossible to evaluate their choice. | *The statistical methods were described for each individual hypothesis in sections 3.4.3 – 3.4.5. This has been clarified by extending the sentence that you mention to “…. using several statistical analyses, which are specified for each hypothesis in the next subsections.” (p.13), and by renumbering sections 3.4.3 – 3.4.5 to 3.4.2.1 – 3.4.2.3 . We have now split the explanation of H2 and H3 from the explanation of H4 and H5. More details are now given for the statical models in the main text (see section 3.4.2). For consistency, we changed some of the statistical test so that they are more closely related to each other, i.e. the models now have a more comparable set-up of fixed effects and interactions. The results and appendix were updated accordingly, but of course this did not change any of the conclusions.*  *Additionally, we repeated in the main text that readers who are interested in all analysis details can find the data analysis code via this project’s Open Science Framework page, see p.8. (“…, and code for data analysis are stored and publicly available at Open Science Framework”) and p.14 (“All data analyses were done in R version 4.2.2 and the R code is available via this project’s Open Science Framework page.”) .* |
| R3.1d | The quantitative results are presented with a few values and a density plot that lack context and completeness: statistics are not mentioned and the value of an observed difference is without a standard deviation and a p-value for statistical significance. | *Your observation is correct and we understand your concerns. We like to explain our motives to present it the way we did: The density plots are included for first insights in the raw data before running any statistical tests. As no statistical tests are done at this point, no statistical tests and p-values are described. It can also be observed that the data is not normally distributed, so reporting only the observed differences and their standard deviations would give less information on the raw data compared to the density plots that show the full distribution. This is the reasons we choose to keep the density plots and explain their interpretation in the text (see e.g. p.23-24 “In Figure 6 we see that in all …. suggesting that the graphs were indeed misleading.“*). |
| R3.1e | The density plot reported in the results section lacks a proper description, even to be addressed qualitatively. | *Thank you for pointing this out. We have added a short explanation of what data a density plot presents in the captions: “Density plots are smoothed versions of histograms that represent the raw distribution of the answers of the participants in the various conditions.”, and we have changed the explanation of the dots in all captions to “condition” instead of “group” as “group” may be confused with Group A and B in the set-up of the study.*  *The observations from the density plots are described in the text.* |
| R3.1f | “*To increase the power, instead of testing for each context, we can combine the graphs and only test for an effect of the misleading graphs within each graph type, which situation is also visualized in Figure 5 (right)*” (p. 21/below figure 5) needs to be better explained addressing pros and cons of your choice. This aspect could be easily fixed by working on the variables presentation. Unfortunately, at this point of the reading the elements mentioned but not explained can hinder proper results reading. | *Thank you for addressing the confusion. You made us realise that the issue of the reduced power due to multiple-testing is not explained well, and that the switch from running multiple t-tests to the mixed effects model can be confusing and is not required to make the point.*  *p.24: We have extended the explanation of the issue of the multiple-testing correction and replaced the mixed effects model with another set of t-tests (again with a correction for multiple testing) which makes it more consistent as it matches the previous analysis and the density plots more closely.  As the statistical test for the influence of graph literacy was an extension of the now removed mixed effects model, we have explained that single model in more detail in the main text.* |
| R3.2 | **Qualitative Methods**  A more detailed description of the methodology with appropriate references would be helpful to better frame the results: “*open and axial coding*” could be detailed with the type of framework you referred to. There are further questionable aspects: were interviews recorded? If not, why not and what was done to ensure the representativeness of the data? Were any strategies employed to ensure data validity? | *Thank you for pointing this out. The interviews were recorded, as is stated on p.11. We have further specified the type of coding with a literature reference (p.13). Appendix SB offers more insight into the process of coding. To ensure data validity, 20% of the data was recoded by a second researcher, as is stated on p.13.* |
| R3.3a | **Exposition**  Although the paper structure is appropriate, the content is not well organized, making it difficult to navigate it smoothly, even within the paragraph. Both the introduction and the literature review could be better structured (e.g. first mention the characteristics you are going to describe and then move on to each one). This type of writing should be as much predictable as possible to make reading easier. | *Thank you for the suggestions. We have accepted your other remark for section 2 (R3.3b), and suggestions from the other reviewers (R1.1, R1.2, R2.3) to improve the readability here.* |
| R3.3b | I would merge paragraphs 2.1, 2.2 and 2.3 in a single one in order to have a stronger context with less lines. | *Suggestion accepted (p.4).* |
| R3.3c | In this connection, I would change 3.1 “*Study Design*”: this creates a kind of Easter egg when you get to the end of the paragraph. Why not say that the initial plan failed due to non collaborative participation and you exploited this experience as a pilot to refine your study design? On the one hand it helped to refine the methodology, but on the other it should not get so much space. | *We see your point of it taking up quite some space. We do however feel it needs to be clearly stated that the first data collection failed and was not originally set up as a pilot study. Also, we feel that the experience of it failing contributes valuable information to the report on this specific group of participants, which is also an important focus of this manuscript.* |
| R3.3d | The description of why you chose this specific target is quite redundant: I would keep it only in the method for just mentioning it in the limitations (see specific note for limitations). | *See R3.3c: challenges we faced while working with this target group is not just a limitation but a substantive part of the manuscript’s focus, and is also mentioned by other reviewers as valuable information. We therefore choose to also keep it in the methods section.* |
| R3.3e | Maybe moving 4.1.1 (*Demographics*) in the sample/participants section: first you describe the population you are interested in and then you present the composition of the sample you recruited. Add mean and standard deviation for age and other measures. | *Thank you for the suggestion. We have added means and SDs (p.18). We choose to keep the table under results to make it easier for readers to look up the participants with the quotes given.* |
| R3.3f | I would suggest better integrating the quotes from interviews to support your argument, even smaller pieces but within the paragraph in the points in which you mention them. | *Thank you for the suggestion. We will consider in consultation with the editor if the quotes should be integrated in the text or kept as they are to stand out.* |
| R3.4 | **Limitations**  Limitations go beyond the generalizability motivated by the specific population targeted. Interactions between the variables should be acknowledged, as well as the potential bias of representation in the topic. I would have expected a more in-depth discussion of the qualitative methodology as well as bias and variables interactions which are pivotal in this study. | *Thank you for your thoughts on the discussion. Indeed a broader discussion would strengthen the paper, however due to word limits we had to make a selection of topics that are most valuable and relevant. Possible bias in the qualitative methodology is discussed for instance on p.29: “The strategy of our participants might have been the result of the instruction to think aloud, assuming that reading out words and numbers is easier than formulating what is seen.”. The issue of variables interactions is now more fully discussed in the Methods section in response to your suggestions under R3.1c. (see section 3.4 in the manuscript).* |
| R3.5 | **Missing Definitions and Descriptions**  Some of the descriptions are too much on a high level making it difficult to be completely sure of what you are referring to. If there are standards or conventions please cite, instead add at least a very short definition. Especially with strong statements, please consider to be more specific. For example “*Hence, from these observations we can conclude that* ***indeed, generally,*** *the misleading bar and pictorial area graphs were misleading, but* ***some contexts*** *resulted in more differences than others.*” (p. 21/below figure 5) is an important conclusion but it lacks completeness. | *We agree that the indicated line is not specific. We have integrated the sentence better with the preceding paragraph that provides the details needed to correctly understand this more general summarizing remark (p.24-25).* |
