# Supplementary material for: Debunking misleading graphs effectively: How vocationally educated young adults perceive graphs
Source: PLoS One. 2026 Feb 9;21(2):e0340100. doi: 10.1371/journal.pone.0340100 (PMC12885246; doi:10.1371/journal.pone.0340100)
Supplement: Attachment — Submitted filename: 251211_Response letter.docx [file pone.0340100.s010.docx]

**Response to editor’s request for revisions**

Manuscript
*Debunking misleading graphs effectively: How vocationally educated young adults perceive graphs.*

| E.0 | Although the study addresses an important and timely topic, the presentation requires substantial revision before the paper can be considered further. | Thank you for appreciating the content of our study report. We are glad to hear Reviewer #1 was pleased with all made adjustments. We are also grateful for the request of Reviewer #4 to work on the structure and readability of the paper. We have taken your suggestions to do so to heart and made changes as reported below. |
| --- | --- | --- |
| E.1 | Substantially condense the manuscript, especially the Introduction and Discussion. Aim to reduce redundancy and remove details that can be streamlined or moved to supplementary material. | We have shortened the manuscript by 1363 words. We condensed long sentences and removed redundant information. We have deleted information in the discussion that is already stated in the supplementary material. |
| E.2 | Reconsider the structure, merging smaller subsections into larger, more integrated sections to improve readability. | In the theoretical framework and methods we have removed repetitive and redundant information, and we removed information that is also discussed elsewhere. We have merged subsections 2.2 and 2.3 into one. |
| E.3 | Sharpen the central message, focusing on the key contribution that misleading graphs can distort perception and that corrective exposure—regardless of design—can mitigate these effects. | In the discussion section, we have removed some issues that lead away from the central message. We hope and believe that the condensation and restructuring of the text contributes to focusing on the central message. |
| E.4 | Present limitations more concisely, for example regarding the challenges of using visual analogue scales, which currently receive extended and somewhat repetitive treatment. | We have removed repetitive treatment of the VAS in the discussion. We downsized the discussion on vocational educated participants to the central issue: their underrepresentation in research. |
